# 伴有*EGFR*突变的非小细胞肺癌血清CYFRA21-1和CEA水平与EGFR-TKIs的疗效关系

**DOI:** 10.3779/j.issn.1009-3419.2016.08.12

**Published:** 2016-08-20

**Authors:** 群慧 王, 华 郑, 范彬 胡, 红梅 张, 瑛 胡, 杰 李, 同梅 张, 赞 刘, 葆华 鲁, 爱民 胡, 宝兰 李

**Affiliations:** 101149 北京, 首都医科大学附属北京胸科医院肿瘤科 Department of Medical Oncology, Beijing Chest Hospital, Capital Medical University/Beijing Tuberculosis and Thoracic Tumor Research Institute, Beijing 101149, China

**Keywords:** 肺肿瘤, 表皮生长因子受体抑制剂, CYFRA21-1, 癌胚抗原, Lung neoplasms, Epidermal growth factor receptor-tyrosine kinase inhibitor, Cytokeratin 19 fragment, Carcinoembryonic antigen

## Abstract

**背景与目的:**

表皮生长因子受体-酪氨酸激酶抑制剂(epidermal growth factor receptor-tyrosine kinase inhibitors, EGFR-TKIs)是*EGFR*基因突变晚期非小细胞癌(non-small cell lung cancer, NSCLC)患者一线标准治疗方案, 但是临床实践中, 疗效差异较大。本项实验拟研究治疗前血清细胞角蛋白19片段(cytokeratin-19 fragments, CYFRA21-1)和癌胚抗原(carcinoembryonic antigen, CEA)的水平是否与EGFR-TKIs疗效有关。

**方法:**

回顾性分析194例*EGFR*基因突变阳性且接受EGFR-TKIs治疗的NSCLC患者的治疗前的血清CYFRA21-1和CEA水平与EGFR-TKIs疗效及生存时间的关系。

**结果:**

血清水平CYFRA21-1增高和正常的无进展生存时间(progression-free survival, PFS)分别为7.0个月和11.9个月(*P* < 0.001);总生存(overall survival, OS)分别为12.6个月和28.0个月(*P* < 0.001)。腺癌中, 血清水平增高和正常的PFS分别为7.0个月和12.0个月(*P* < 0.001), OS分别为13.1个月和28.1个月(*P* < 0.001)。鳞癌中, 血清CYFRA21-1水平高低与生存时间无关。治疗前血清CEA水平高低与生存时间无关。

**结论:**

在*EGFR*突变肺腺癌患者, 治疗前血清水平CYFRA21-1增高组EGFR-TKIs治疗的PFS和OS均较正常组短。*EGFR*突变肺腺癌患者, 治疗前血清CYFRA21-1水平可以作为预测EGFR-TKIs治疗的疗效指标。

## Abstract (en)

**Background and objective:**

Epidermal growth factor receptor-tyrosine kinase inhibitors (EGFR-TKIs) are the standard first-line treatment regimen for *EGFR* mutated non-small cell lung cancer (NSCLC) patients.However, the efficacy of EGFR-TKIs widely varies.The aim of this study is to determine whether the pretreatment serum cytokeratin-19 fragments (CYFRA21-1) and carcinoembryonic antigen (CEA) are associated with the efficacy of EGFR-TKIs in *EGFR*-mutated NSCLC patients.

**Methods:**

We retrospectively enrolled 194 NSCLC patients harboring *EGFR* mutations who received EGFR-TKIs.Clinical characteristics were collected, and the relation between the efficacy of EGFR-TKIs and pretreatment serum CYFRA21-1 and CEA was analyzed.

**Results:**

In all cases, progression-free survival (PFS) in patients with high CYFRA21-1 level was significantly shorter than PFS in patients with normal CYFRA21-1 (7.0 *vs* 11.9 months, *P* < 0.001).Overall survival (OS) in patients with high CYFRA21-1 was significantly shorter than in the normal-CYFRA21-1 group (12.6 *vs* 28.0 months, *P* < 0.001).In adenocarcinoma patients, PFS in the high-CYFRA21-1 level group was significantly shorter than in patients with normal CYFRA21-1 (7.0 *vs* 12.0 months, *P* < 0.001).OS in patients with high CYFRA21-1 was significantly shorter than that in the normal-CYFRA21-1 group (13.1 *vs* 28.1 months, *P* < 0.001).Among squamous carcinoma patients, CYFRA21-1 level did not affect survival.No significant difference in PFS and OS was observed between patients with high CEA and patients with normal CEA.

**Conclusion:**

*EGFR*-mutated patients with high CYFRA21-1 had significantly shorter PFS and OS than patients with normal CYFRA21-1 after receiving EGFR-TKIs.Pretreatment serum CYFR21-1 level was a predictive marker of EGFR-TKI treatment in *EGFR*-mutated NSCLC patients.

肺癌是全世界发病率和死亡率最高的恶性肿瘤, 5年生存率仅为16.8%^[1]^。非小细胞癌(non-small cell lung cancer, NSCLC)约占所有肺癌病理类型85%, 而且 > 70%的患者确诊都是晚期^[2]^。全身化疗一直是这部分人群主要治疗方法。21世纪初随着分子生物学研究不断深入, 开启了NSCLC以表皮生长因子受体(epidermal growth factor receptor, *EGFR*)基因突变为指导的靶向治疗时代, 而IPASS、NEJGSG、WJTOG3405、OPTIMAL、EURTAC、LUX-Lun3及ICOGEN等研究, 进一步确立表皮生长因子受体-酪氨酸激酶抑制剂(epidermal growth factor receptor-tyrosine kinase inhibitors, EGFR-TKIs)在*EGFR*基因突变的NSCLC晚期患者治疗地位^[3-9]^。

血清癌胚抗原(carcinoembryonic antigen, CEA)和细胞角蛋白19片段(cytokeratin-19 fragments, CYFRA21-1)是最常见肿瘤标志物, 应用于肺癌的诊断、预后和监测。目前认为CEA是肺腺癌较好的肿瘤标志物, 而CYFRA21-1则是肺鳞癌诊断的最好肿瘤标志物。本文回顾性研究伴有*EGFR*基因的突变NSCLC晚期患者治疗前血清CEA和CYFRA21-1的水平和EGFR-TKIs疗效及预后的关系, 希望给临床EGFR-TKIs靶向治疗的个体化提供参考依据。

## 1 资料与方法

### 1.1 对象

本研究收集首都医科大学附属北京胸科医院于2010年10月-2015年3月*EGFR*基因突变且应用EGFR-TKIs治疗的患者194例, 所有患者均通过病理及免疫组化诊断为NSCLC, 并且为Ⅲb期-Ⅳ期或者手术后复发的晚期患者。患者年龄30岁-85岁(中位年龄58岁), 男性86例(44.3%), 女性108例(55.7%)。PS评分0分-1分166例(85.6%), 2分-4分28例(14.4%)。吸烟67例(34.5%), 非吸烟127例(65.5%)。IIIb期患者6例(3.1%), Ⅳ期188例(96.9%), 中心型26例(13.4%), 周围型168例(86.6%)。腺癌179例(92.3%), 鳞癌9例(4.6%), 非小细胞癌3例(1.5%), 腺鳞癌2例(1%), 大细胞癌1例(0.5%)。患者的临床特征见表 1。

**1:** 194例携带*EGFR*突变的NSCLC患者的临床特征 Characteristics of the 194 NSCLC patients harboring *EGFR* mutations

Characteristics	No.of patients	Percentage(%)
Age (yr)		
≤70	165	85.1
> 70	29	14.9
Gender		
Male	86	44.3
Female	108	55.7
Histology		
Adenocarcinoma	179	92.3
Squamous	9	4.6
Others	6	3.1
PS		
0-1	166	85.6
2-4	28	14.4
Staging		
Ⅲb	6	3.1
Ⅳ	188	96.9
Smoking history		
Smoking	67	34.5
Non-smoking	127	65.5
EGFR gene		
Exton 19 (19del)	112	57.7
Exton 21 (L858R)	72	37.1
Others	10	5.2
EGFR-TKIs		
Icotinib	115	59.3
Gefitinib	44	22.7
Erlotinib	35	18
Therapy		
First-line	117	60.3
Second-line	65	33.5
Third-line	9	4.6
Fourth-line	3	1.5
EGFR-TKIs:epidermal growth factor receptor-tyrosine kinase inhibitors; NSCLC:non-small cell lung cancer; PS:performance status.		

### 1.2 治疗方法

所有患者都给予埃克替尼或者吉非替尼、厄洛替尼治疗。埃克替尼125 mg, 每日3次口服; 吉非替尼250 mg, 每日1次; 厄洛替尼150 mg每日1次。接受埃克替尼治疗115例(59.3%), 吉非替尼44例(22.7%), 厄洛替尼35例(18%)。

### 1.3 *EGFR*基因检测

采取两种方法:一种PCR-Sanger测序法, 由北京海思特临床检验所检测; 一种ARMS荧光定量PCR, 检测试剂为厦门艾德人类*EGFR*基因突变检测试剂盒。应用PCR-Sanger测序法123例(63.4%), ARMS法71例(36.6%)。标本来源:气管镜活检47例(24.2%), 肺穿刺活检88例(45.4%), 胸水沉淀包埋31例(16%), 手术标本13例(6.7%), 淋巴结活检14例(7.2%), 骨转移穿刺1例(0.5%)。*EGFR*基因检测结果:19外显子缺失突变112例(57.7%), 21外显子错义突变72例(37.1%), 少见突变3例(G719X)(1.5%), 伴有原发T790m突变1例(0.5%), *EGFR*基因突变2例(1.0%), 伴有*Kras*突变2例(1.0%), 19外显子错义突变2例(1.0%)。

### 1.4 血清CEA和CYFRA21-1的检测

EGFR-TKIs治疗前清晨空腹静脉血, 分离血清后, 使用上海透景生命科技股份有限公司试剂盒及Luminex多功能流式点阵仪应用流式荧光发光法进行测定。根据我院试剂盒正常参考值进行结果判定:CEA≤6 ng/mL为正常, CEA > 6 ng/mL为表达水平增高; CYFRA21-1≤6 ng/mL为正常, CYFRA21-1 > 6 ng/mL为表达水平增高。

### 1.5 疗效评定及生存指标

治疗前2周对疾病状况进行评估, 治疗后4周复查。以后每2个月-3个月随访1次。按实体瘤疗效评价标准(Response Evaluation Criteria in Solid Tumors, RECIST)评价近期疗效, 分为完全缓解(complete response, CR)、部分缓解(partial response, PR)、疾病稳定(stable disease, SD)和疾病进展(progressive disease, PD)。生存指标为无进展生存时间(progression-free survival, PFS)定义为EGFR-TKI治疗开始至疾病进展或未进展死亡的时间。总生存(overall survival, OS)定义为EGFR-TKI治疗开始到死亡或末次随访时间。

### 1.6 统计学方法

使用SPSS V22.0软件, 采用*Kaplan-Meier*法并进行*Log-rank*检验生存分析, 用*Cox*比例风险模型进行多因素分析, 所有统计结果以*P* < 0.05为差异有统计学意义。所有患者均随访至2015年12月31日, 其中死亡103例, 存活90例, 1例失访。死亡病例为截尾数据, 存活病例为未截尾数据。

## 2 结果 

### 2.1 EGFR-TKIs疗效及多因素生存分析

本研究中患者的总体疗效:CR、PR、SD、DCR及PD分别为0.5%(1例)、68.6%(133例)、24.2%(47例)、93.3%(181例)和6.7%(13例)。中位PFS为9.0个月(95%CI:7.3-10.7);中位总生存OS为23.0个月(95%CI:20.2-25.8)。单因素生存分析显示:年龄 > 70和年龄≤70岁PFS分别为9.0个月和10.5个月(*P*=0.495);OS分别为23.0个月和24个月(*P*=0.441);男性和女性PFS分别为10.5个月和8.2个月(*P*=0.391);OS分别为24.0个月和21.8个月(*P*=0.717);吸烟和非吸烟PFS分别为10.5个月和8.9个月(*P*=0.406);OS分别为24.1个月和21.8个月(*P*=0.886);19外显子缺失突变和21外显子错义突变PFS分别为9.0个月和8.2个月(*P*=0.375);OS分别为24.1个月和17.2个月(*P*=0.143);以上单因素分析均无统计学差异。PS评分0分-1分和2分-4分PFS分别为10.5个月和5.3个月(*P* < 0.001);OS分别为24.8个月和9.7个月(*P* < 0.001);腺癌和鳞癌PFS分别为9.0个月和4.1个月(*P*=0.009);OS分别为23.1个月和8.1个月(*P* < 0.001);治疗前基线无脑转移和伴有脑转移PFS分别为10.9个月和7.5个月(*P*=0.001);OS分别为26.1个月和14.3个月(*P* < 0.001);无肝转移和伴有肝转移PFS分别为10.2个月和7.0个月(*P*=0.002);OS分别为23.7个月和12.0个月(*P*=0.008)。上述单因素分析有统计学差异。多因素生存分析显示PS评分状态和是否伴有脑转移PFS和OS都有统计学意义。而组织类型中腺癌和鳞癌PFS无统计学差异, 但在OS还是有统计学差异(表 2, 表 3)。

**2:** 单因素生存分析 Factor associated with PFS and OS

Factor	*n*	Median PFS (m)	*P*	Median OS (m)	*P*
Age (yr)			0.495		0.441
≤70	165	9.0		23.0	
> 70	29	10.5		24.0	
Gender			0.391		0.717
Male	86	10.5		24.0	
Female	108	8.2		21.8	
Smoking hisory			0.406		0.886
Smoking	67	10.5		24.1	
Non-smoking	127	8.9		21.8	
PS			< 0.001		< 0.001
0-1	166	10.5		24.8	
2-4	28	5.3		9.7	
Histology			0.009		< 0.001
Adenocarcinoma	179	9.0		23.1	
Squamous	9	4.1		8.1	
*EGFR* gene			0.375		0.143
Exton 19 (19del)	112	9.0		24.1	
Exton 21 (L858R)	72	8.2		17.2	
CEA			0.294		0.122
≤6 ng/mL	69	10.2		24.0	
> 6 ng/mL	125	8.9		21.8	
CYFRA21-1			< 0.001		< 0.001
≤6 ng/mL	129	11.9		28.0	
> 6 ng/mL	65	7.0		12.6	
Distant metastases					
Cerebral metastases	58	7.5	0.001	14.3	< 0.001
Non-cerebral metastases	136	10.9		26.1	
Hepatic metastases	19	7.0	0.002	12.0	0.008
Non-hepatic metastases metastases	175	10.2		23.7	
EGFR-TKIs			0.954		0.465
Lcotinib	115	8.2		21.1	
Gefitinib	44	9.0		23.0	
Erlotinib	35	11.0		24.8	
Method			0.868		0.159
PCR-Sanger	123	9.0		21.1	
ARMS	71	10.2		23.0	
PFS:progression-free survival; OS:overall survival.					

**3:** 多因素生存分析 Multivariate analysis of PFS and OS

Factor	Median PFS			Median OS	
	HR (95%CI)	*P*		HR (95%CI)	*P*
PS (0-1 *vs* 2-4)	0.53 (0.34-0.82)	0.005		0.33 (0.19-0.54)	< 0.001
Histology (adenocarcinoma *vs* squamous)	0.69 (0.33-1.40)	0.301		0.46 (0.22-0.97)	0.042
CEA (≤6 ng/mL *vs* > 6 ng/mL)	0.86 (0.61-1.22)	0.394		0.72 (0.47-1.11)	0.138
CYFRA21-1 (≤6 ng/mL *vs* > 6 ng/mL)	0.62 (0.44-0.87)	0.006		0.30 (0.19-0.47)	< 0.001
Non *vs* Cerebral metastases	0.63 (0.45-0.89)	0.009		0.45 (0.29-0.68)	< 0.001
Non *vs* Hepatic metastases	0.64 (0.37-1.09)	0.100		0.82 (0.44-1.53)	0.533

### 2.2 治疗前血清CYFRA21-1水平与PFS、OS的相关性分析

194例患者中129例血清水平CYFRA21-1正常, 65例血清CYFRA21-1水平增高, 血清水平CYFRA21-1正常和增高的PFS分别为11.9个月和7.0个月(*P* < 0.001), OS分别为28.0个月和12.6个月(*P* < 0.001), 均有统计学差异(表 2)。亚组分析中, 腺癌组120例血清CYFRA21-1水平正常, 59例血清水平增高, 它们的PFS分别为12.0个月和7.0个月(*P* < 0.001), OS分别为28.1个月和13.1个月(*P* < 0.001)均有统计学差异(表 4)。多因素分析中(表 3), PFS(*P*=0.006, HR=0.62, 95%CI:0.44-0.87)(图 1), OS(*P* < 0.001, HR=0.30, 95%CI:0.19-0.47)(图 2), 也均有统计学差异。鳞癌组4例血清水平CYFRA21-1正常, 5例血清水平增高, 它们的PFS分别为4.1个月和3.1个月(*P*=0.529), OS分别为8.1个月和7.0个月(*P*=0.359), 均无统计学差异。

**1:**
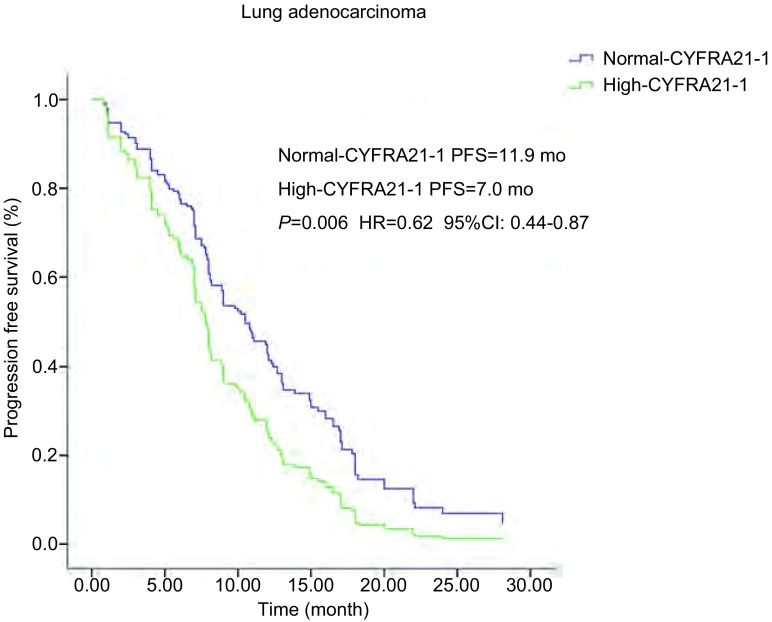
肺腺癌患者血清CYFRA21-1水平和PFS生存曲线关系。血清CYFRA21-1水平正常和增高PFS分别为12.0个月和7.0个月(*P*=0.006, HR=0.62, 95%CI:0.44-0.87)。 *Kaplan-Meier* survival curves of progression-free survival (PFS) according to serum CYFRA21-1 level in lung adenocarcinoma patients.PFS in normal-and high serum CYFRA21-1 level was 12.0 months and 7.0 months, respectively (*P*=0.006, HR=0.62, 95%CI:0.44-0.87).

**2:**
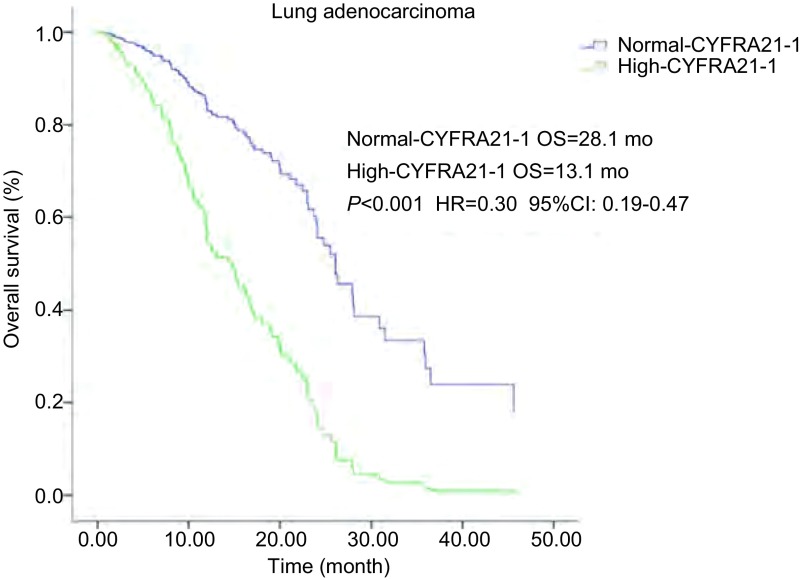
肺腺癌患者血清CYFRA21-1水平和OS生存曲线关系。血清CYFRA21-1水平正常和增高OS分别为28.1个月和13.1个月(*P* < 0.001, HR=0.30, 95%CI:0.19-0.47)。 *Kaplan-Meier* survival curves of overall survival (OS) according to serum CYFRA21-1 level in lung adenocarcinoma patients.OS in normal-and high serum CYFRA21-1 level was 28.1 months and 13.1 months, respectively (*P* < 0.001, HR=0.30, 95%CI:0.19-0.47).

### 2.3 治疗前血清CEA水平与PFS和OS的相关性分析

血清CEA水平正常69例, 血清CEA水平增高125例。它们的PFS分别为10.2个月和8.9个月(*P*=0.294), OS分别为24.0个月和21.8个月(*P*=0.122)均无统计学差异。腺癌亚组血清CEA水平正常及增高的PFS均为9.0个月(*P*=0.436), 无统计学差异(图 3); OS分别为24.8个月和23.0个月(*P*=0.104), 无统计学差异(图 4)。鳞癌亚组血清CEA水平正常2例, 血清水平增高7例; 它们的PFS分别为7.8个月和3.1个月(*P*=0.103), 无统计学差异; OS分别为9.1个月和7.0个月(*P*=0.381), 无统计学差异(表 2-表 4)。

**3:**
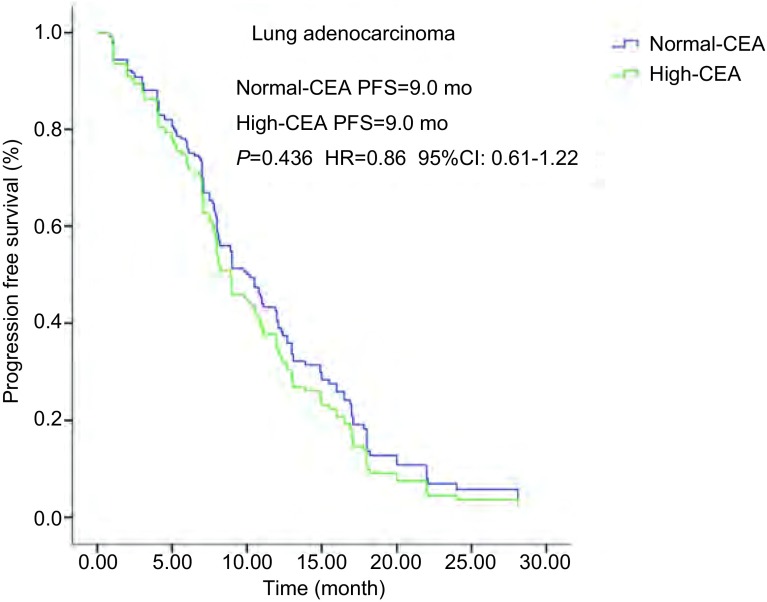
肺腺癌患者血清CEA水平和PFS生存曲线关系。血清CEA水平正常和增高PFS均为9.0个月(*P*=0.436, HR=0.86, 95%CI:0.61-1.22)。 *Kaplan-Meier* survival curves of progression-free survival (PFS) according to serum CEA level in lung adenocarcinoma patients.PFS in normal-and high serum CEA level both were 9.0 months (*P*=0.436, HR=0.86, 95%CI:0.61-1.22).

**4:**
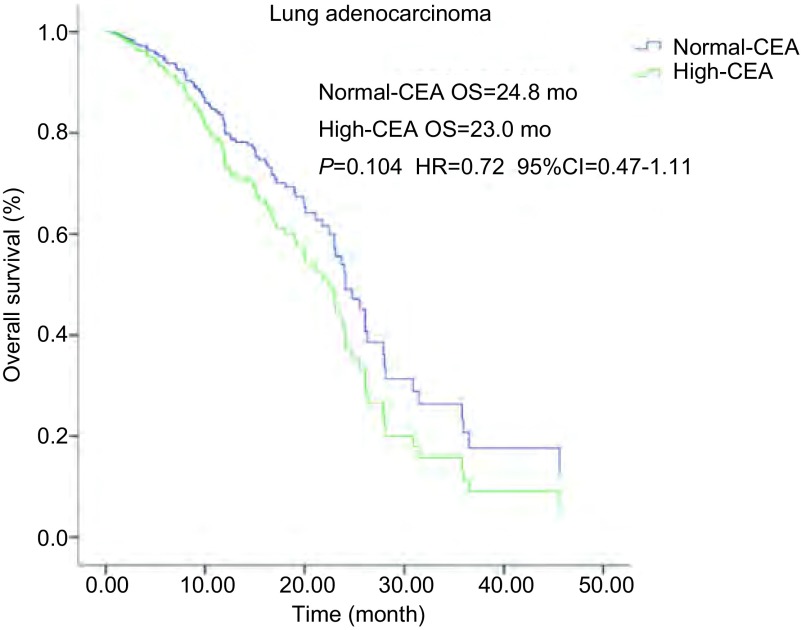
肺腺癌患者, 血清CEA水平和OS生存曲线关系。血清CEA水平正常和增高OS分别为24.8个月和23.0个月(*P*=0.104, HR=0.72, 95%CI:0.47-1.11)。 *Kaplan-Meier* survival curves of overall survival (OS) according to serum CEA level in lung adenocarcinoma patients.OS in normal-and high serum CEA level was 24.8 months and 23.0 months, respectively (*P*=0.104, HR=0.72, 95%CI:0.47-1.11).

**4:** 血清CEA、CYFRA21-1水平亚组生存分析 Subset analysis of PFS and OS

Factor		*n*	Median PFS(mo)	*P*	Median OS(mo)	*P*
Adenocarcinoma	CEA			0.436		0.104
	≤6 ng/mL	64	9.0		24.8	
	> 6 ng/mL	115	9.0		23.0	
	CYFRA21-1			< 0.001		< 0.001
	≤6 ng/mL	120	12.0		28.1	
	> 6 ng/mL	59	7.0		13.1	
Squamous	CEA			0.103		0.381
	≤6 ng/mL	2	7.8		9.1	
	> 6 ng/mL	7	3.1		7.0	
	CYFRA21-1			0.529		0.359
	≤6 ng/mL	4	4.1		8.1	
	> 6 ng/mL	5	3.1		7.0	

## 3 讨论

2004年Lynch等^[10]^研究发现*EGFR*基因突变状态与EGFR-TKIs的疗效相关, 并且近几年多项国际多中心的临床研究已显示EGFR-TKIs在*EGFR*基因突变的NSCLC晚期患者有着非常好的疗效及低毒性^[3-9]^。2012年在美国国立综合癌症网络(National Comprehensive Cancer Network, NCCN)NSCLC临床实践指南推荐对于*EGFR*基因突变的晚期, 复发或转移的NSCLC患者EGFR-TKIs作为一线治疗。随着对EGFR-TKIs进一步研究, 发现同样有*EGFR*基因突变, 但不同的临床特征, EGFR-TKIs疗效是有差异的。Lee等^[11]^*meta*分析中认为女性, 非吸烟患者, 腺癌及19外显子缺失突变, 相对疗效更好一些。我们的研究显示, 性别、年龄、吸烟状态及*EGFR*基因敏感突变类型之间无统计学差异。但是PS评分0分-1分较2分-4分疗效更好, 有统计学差异; 腺癌较鳞癌患者EGFR-TKIs疗效更好, 有统计学差异。在合并有远处转移, 有脑转移和肝转移的患者PFS和OS均有缩短。多因素生存分析显示:PS状态差, 鳞癌和伴有脑转移的患者预后差, OS有统计学意义。

CYFRA21-1是我们临床实践中肺癌诊断常用肿瘤标志物。当肿瘤细胞发生溶解时, 其中的细胞角质蛋白释放入血, 而使血中CYFRA21-1升高。其对肿瘤的诊断敏感性约为50%, 且以鳞癌敏感性最高。有报道其对鳞癌的敏感性和特异性分别为66.5%和95%^[12]^, 因此众多学者一致认为CYFR21-1是鳞癌鉴别诊断的最好肿瘤标志物, 其水平高低和敏感度与病情呈正相关。近年Tanaka等^[13]^提出伴有*EGFR*突变的NSCLC, 治疗前血清CYFRA21-1水平是预测EGFR-TKIs疗效的指标, 但相关报道国内外还是比较少。我们的研究显示治疗前血清水平CYFRA21-1正常和增高的PFS分别为11.9个月和7.0个月(*P* < 0.001), OS分别为28.0个月和12.6个月(*P* < 0.001), 均有差异。亚组分析中, 腺癌组血清CYFRA21-1水平正常和增高的PFS分别为12.0个月和7.0个月(*P* < 0.001), OS分别为28.1个月和13.1个月(*P* < 0.001)均有差异。多因素分析中, PFS(*P*=0.006, HR=0.62, 95%CI:0.44-0.87), OS(*P* < 0.001, HR=0.30, 95%CI:0.19-0.47)也均有统计学差异。鳞癌组血清水平CYFRA21-1正常和增高的PFS分别为4.1个月和3.1个月(*P*=0.529), OS分别为8.1个月和7.0个月(*P*=0.359), 均无差异。我们研究显示伴有*EGFR*基因突变的肺腺癌患者, 治疗前血清CYFRA21-1水平增高较正常水平相比较, PFS和OS都有缩短; 治疗前血清CYFRA21-1水平可以预测EGFR-TKIs疗效及预后。而肺鳞癌在我们研究中由于病例数较少, 并未显示治疗前血清CYFRA21-1水平与EGFR-TKIs的疗效和预后的关系。

*EGFR*基因突变, 在肺腺癌患者比较常见, PIONEER临床试验显示东亚肺腺癌患者50.2%的*EGFR*基因的突变率^[14]^。而肺鳞癌患者*EGFR*基因的突变比较少见, Dearden等^[15]^*meta*分析中, 东亚肺鳞癌患者*EGFR*基因的突变率仅为4.6%, 而中国报道肺鳞癌患者*EGFR*基因的突变则在14%-25%^[16-18]^。并且在既往的报道*EGFR*基因突变肺鳞癌患者采用EGFR-TKIs治疗的PFS和OS明显低于肺腺癌, PFS在3个月-7个月, OS在9.4个月-14.7个月^[19-21]^。本研究中*EGFR*基因的突变患者鳞癌患者9例, 为4.6%。与肺腺癌相比PFS分别为4.1个月和9.0个月(*P*=0.009), OS分别为8.1个月和23.1个月(*P* < 0.001)。肺鳞癌*EGFR*突变患者临床受益程度明显低于*EGFR*突变肺腺癌患者, 似乎提示肺鳞癌中具有*EGFR*突变基因也许并不是靶向治疗的“驱动基因”, 可能存在其他肿瘤驱动机制。肺癌的组织类型较多, 且以腺癌的组织分型最复杂, 异质性最明显。Travis等^[22]^认为由于肿瘤组织的异质性, 基于小标本的病理组织诊断是有限, 有可能会带来相反的组织类型诊断。并且由于肿瘤组织的异质性, 小标本病理活检有可能未体现出整体的病理组织类型状态。我们的研究患者均为Ⅲb期-Ⅳ期晚期患者, 标本来源:气管镜活检47例(24.2%), 肺穿刺活检88例(45.4%), 胸水沉淀包埋31例(16%), 手术标本13例(6.7%), 淋巴结活检14例(7.2%), 骨转移穿刺1例(0.5%), 绝大部分是小标本的病理活检。肺腺癌患者血清水平CYFRA21-1异常增高, 我们考虑由于肿瘤异质性, 可能混有鳞癌的成份, 而肺鳞癌较肺腺癌相比较, EGFR-TKIs疗效不佳, 那么治疗前血清CYFRA21-1水平异常增高可能带来EGFR-TKIs治疗的不佳疗效。而我们研究也显示伴有*EGFR*基因突变的肺腺癌者, 治疗前血清CYFRA21-1水平增高, PFS和OS都短, 血清CYFRA21-1水平可能是预测EGFR-TKIs治疗的疗效及预后的指标。

CEA是最早发现, 目前临床应用最广的一种肿瘤抗原, 在成人肺、乳腺和胃肠等腺癌组织有表达。肺癌细胞可合成和释放CEA, 目前认为CEA是肺癌尤其是腺癌的进展、疗效和预后评估较好的一个肿瘤标志物^[23]^。与血清CYFRA21-1不同, 近年的相关报道显示治疗前血清CEA水平并不能预测EGFR-TKIs治疗的疗效。我们的研究显示治疗前血清CEA水平正常和增高的PFS分别为10.2个月和8.9个月(*P*=0.294), OS分别为24.0个月和21.8个月(*P*=0.122)均无统计学差异。腺癌亚组血清CEA水平正常及增高的PFS均为9.0个月(*P*=0.436), 无统计学差异; OS分别为24.8个月和23.0个月(*P*=0.104), 无统计学差异。鳞癌亚组血清CEA水平正常和增高的PFS分别为7.8个月和3.1个月(*P*=0.103), OS分别为9.1个月和7.0个月(*P*=0.381), 均无统计学差异。这些表明治疗前血清CEA水平与伴有*EGFR*突变的NSCLC的EGFR-TKIs治疗的疗效无关, 并不能预测EGFR-TKIs治疗的疗效。

我们的研究显示, 肺腺癌中治疗前血清水平CYFRA21-1增高与正常相比较EGFR-TKIs治疗PFS和OS都有缩短, 而国外相关研究报道治疗前血清水平CYFRA21-1增高的患者EGFR-TKIs治疗仅有PFS缩短, 因为进展后的相关治疗, 两组总的OS是无统计学差异的。那么在我们的研究中出现的OS的显著性缩短, 分析其原因可能是, 我们研究中194例患者中有159例出现进展, 而42例(26.4%)患者随后未再进行化疗、放疗或者其他的靶向药物治疗, 而仅仅给予姑息维持治疗, 从而导致OS进一步缩短。说明在我们临床实践中初次的EGFR-TKIs治疗明显影响OS。NCCN指南中, 对于伴有*EGFR*突变的NSCLC的推荐单药EGFR-TKIs一线治疗, 但关于如何延长EGFR-TKIs治疗PFS的探索性研究近年不断涌现。FASTACTII、NEJ005及JO2557等^[24-26]^多中心的临床研究显示, EGFR-TKIs和化疗交替应用, 或者联合化疗药物, 抗血管药物可以延长PFS至15个月-16个月。但由于临床研究设计的局限性或两药联合应用增加的副反应, 给这些研究带来许多争议。那么对于肺腺癌*EGFR*基因突变患者, 治疗前血清CYFRA21-1水平异常增高且预测单药EGFR-TKIs可能疗效不佳, 给予EGFR-TKIs和化疗交替应用或者联合化疗药物, 是否能延长这部分人群PFS, 从而提高OS, 这还需要将来临床实验进一步探索研究。

我们的研究存在许多局限性和不足, 首先这是个回顾研究, 随访时间2个月-3个月随访一次; 并且*EGFR*基因突变的检测方法没有统一, 而是采取两种方法, PCR-Sanger测序法和ARMS荧光定量PCR法, 但两种方法PFS和OS都没有统计学差异; 我们的研究应用EGFR-TKIs药物也不是统一, 包括有埃克替尼、吉非替尼和厄洛替尼, 但是在我们的研究里, 这三个EGFR-TKI药物无论PFS和OS也都没有统计学差异。虽然有许多局限性和不足, 但是从我们的研究中可以看到伴有*EGFR*突变的NSCLC的EGFR-TKIs治疗疗效还是有许多差异, 提示我们可以针对特定人群给予个体化治疗。

综上所述, 我们研究的结论是:伴有*EGFR*突变的NSCLC的EGFR-TKI治疗中, PS状态差、鳞癌和伴有脑转移的患者EGFR-TKIs治疗预后差。小标本病理有其局限性, 有时是不能代表整体病理组织类型。伴有*EGFR*突变的肺腺癌患者, 治疗前血清水平CYFRA21-1增高者与正常者相比, EGFR-TKIs治疗的PFS和OS均有缩短, 治疗前血清CYFRA21-1水平可以作为预测EGFR-TKIs疗效指标, 也可能是EGFR-TKIs治疗的预后指标; 而治疗前血清CEA水平则不能预测EGFR-TKIs疗效。
